# Epidemiological data on nutritional disorders and outcomes in hospitalized Thai children: an analysis of data from the National Health Database 2015-2019

**DOI:** 10.4178/epih.e2022047

**Published:** 2022-05-16

**Authors:** Suchaorn Saengnipanthkul, Jeeraparn Phosuwattanakul, Kaewjai Thepsuthammarat, Nalinee Chongviriyaphan

**Affiliations:** 1 Division of Nutrition, Department of Pediatrics, Faculty of Medicine Srinagarind Hospital, Khon Kaen University, Khon Kaen, Thailand; 2 Division of Nutrition, Department of Pediatrics, Faculty of Medicine Ramathibodi Hospital, Mahidol University, Bangkok, Thailand; 3 Epidemiology Unit, Faculty of Medicine, Khon Kaen University, Khon Kaen, Thailand

**Keywords:** Children, Hospitalization, Malnutrition, Nutritional disorders, Prevalence

## Abstract

**OBJECTIVES:**

Malnutrition in hospitalized patients is a frequently overlooked health issue. We aimed to assess the prevalence and pattern of nutritional disorders in hospitalized Thai children from the National Health Database.

**METHODS:**

Hospitalized children aged 1 month to 18 years diagnosed with nutritional disorders between 2015 and 2019 were retrospectively reviewed using the National Health Security Office data. Based on the International Classification of Diseases, 10th revision, Clinical Modification, nutritional disorders were classified into 3 major forms of malnutrition: undernutrition (E40-E46), overweight and obesity (E66), and micronutrient deficiencies (D50-D53, E50-E56, E58, E60-E61, and E63).

**RESULTS:**

Out of 5,188,033 hospitalized children, malnutrition was identified in 115,254 (2.2%). Protein-energy malnutrition (PEM), overweight and obesity, and micronutrient deficiencies were prevalent in 0.21%, 0.27%, and 1.81%, respectively. Among those with micronutrient deficiencies, 95.0% had iron deficiency anemia, 2.2% had vitamin D deficiency, and 0.7% had zinc deficiency. Children aged under 5 years mostly had PEM, followed by iron deficiency anemia. Teenagers commonly had obesity and vitamin D deficiency. Patients with PEM who were admitted with common diseases had significantly longer hospital stays and higher hospital costs and mortality rates than those without PEM.

**CONCLUSIONS:**

Hospitalized children had various nutritional disorders, particularly PEM, which was associated with higher morbidity and mortality. Nutritional screening tools should be utilized for the early detection and treatment of malnutrition. Specific International Classification of Diseases codes for nutritional care services and intervention should be available. Additionally, nutritional interventions should be reimbursed, along with nutritional education and empowerment of healthcare providers, to improve hospital care service and improve patient outcomes.

## INTRODUCTION

Malnutrition is a common problem among hospitalized children that may occur either before or during hospitalization. Malnourished patients suffer from immune system deterioration or malfunction, which results in an increased infection rate, impaired immune response, delayed wound healing, and delayed recovery [1,2]. Moreover, malnutrition in hospitalized patients also causes a significant financial burden. Its prevalence varies among studies according to the diagnostic criteria and hospital setting, with reported values ranging from 6% to 50% in developing and developed countries [3]. In 2019, we conducted a multicenter study and found that the prevalence of underweight, wasting, stunting, and overweight and obesity in hospitalized pediatric patients was 21%, 16%, 24%, and 10%, respectively [4].

Malnutrition is a health condition marked by inadequate, unbalanced, or excessive consumption of macronutrients and/or micronutrients, causing adverse effects on growth, physiological functions, and clinical outcomes. It can be broadly categorized into 3 conditions: (1) undernutrition (wasting [low weight-for-length or weight-for-height], stunting [low length- or height-for-age], and underweight [low weight-for-age]); (2) overnutrition (overweight and obesity); and (3) micronutrient deficiencies (vitamin and/or mineral deficiency) [5]. This study focused on all forms of malnutrition, which were referred to as “nutritional disorders,” in hospitalized Thai children according to the World Health Organization’s definition.

Thailand’s sources of reimbursement funds are the government’s three healthcare schemes: the Universal Coverage (UC) scheme (administered by the National Health Security Office, NHSO), the Social Security scheme (under the Social Security Office), and the Civil Servant Medical Benefits (run by the Comptroller General’s Department). The UC scheme is the major health financing scheme covering, approximately 75% of healthcare reimbursements in the Thai population. Its reimbursement system uses the diagnosis-related group (DRG) method for payments [6].

In this study, we aimed to determine the prevalence of nutritional disorders among hospitalized Thai children and to identify the association between undernutrition and hospital outcomes in tertiary-care hospitals.

## MATERIALS AND METHODS

Hospitalized children between October 1, 2014 and September 30, 2019 (5 fiscal years, 2015-2019) were retrospectively analyzed using the data from the NHSO in Thailand. We included hospitalized children aged 1 month to 18 years, with a coded diagnosis of nutritional disorder (CND) defined by selected International Classification of Diseases, 10th revision, Clinical Modification (ICD-10-CM) codes. Therefore, these pediatric patients were categorized into the following CND subgroups: protein-energy malnutrition (PEM) (E40-E46), overweight or obesity (E66), and micronutrient deficiencies (D50-D53, E50-E56, E58, E60-E61, and E63) (Supplementary Material 1). The presence of 1 or more of the codes, as either the principal diagnosis or comorbidities, was defined as inpatient CND. Considering that the NHSO data contain personal identifiers, we could identify the hospitalizations of the same patient. The unit of analysis to demonstrate the prevalence of nutritional disorders was presented in the case.

Between 2015 and 2019, the NHSO covered approximately 13 million children aged 1 month to 18 years annually. The male-to-female ratio was 1:1, and the age group with the highest prevalence was 5 years to under 13 years (40%), followed by 1 year to under 5 years (26%). One-third of the children lived in the Northeast region. The UC schemes recorded 8,993,210 hospitalizations from 5,188,033 patients (i.e., approximately 1.3-1.4 million cases per year).

To determine the burden of PEM and the association between PEM and hospital outcomes, we further analyzed the data of patients with a coded diagnosis of PEM in tertiary-care hospitals.

Nutritional disorders may be under-recognized and under-documented, thereby underestimating a real health issue. Thus, we included data from tertiary-care hospitals with a dedicated nutritional care team. Given that individual patients could have been admitted with different principal diagnoses during the study period of 5 fiscal years, the unit of analysis was per hospitalization, not per case. The outcome variables were the length of hospital stay (LOS), total hospital costs, and mortality rates. The LOS was defined as the number of days between the date of admission and discharge. The hospital cost was the inpatient-care cost based on the Thai DRG system. The mortality rate referred to the number of patients who died before discharge. Additionally, we assessed the burden of PEM as a comorbidity in patients admitted with common medical conditions as the principal diagnosis. These common medical diagnoses included respiratory infections (J00-J22, J40-J47, J85-J86), intestinal infections (A00-A09), digestive system disorders (K00-K93, R10-R19), and arthropod-borne viral fever (A75-A79, A90-A99). The association between PEM and neoplasms (C00-C97, D00-D48) was also investigated as a specific topic of interest related to chronic medical conditions in this study.

Data were exported to Stata version 10.1 (StataCorp., College Station, TX, USA). Percentages, medians, and interquartile ranges were used as descriptive statistics. The rates of selected clinical comorbidities and hospital outcomes (median LOS, total cost, and in-hospital mortality) were compared between children with and without PEM code diagnoses. Categorical and continuous variables were compared using the Fisher exact test and the Wilcoxon rank-sum test, respectively. The cut-off for statistical significance was set to p< 0.05.

### Ethics statement

The Royal College of Pediatricians of Thailand and the Pediatric Nutrition Association of Thailand with the NHSO approved this study. The study protocol was approved by the Khon Kaen University Ethics Committee for Human Research (HE631410). The requirement for informed consent was waived by the ethics committee.

## RESULTS

### Prevalence of hospitalized children with a coded diagnosis of nutritional disorder (CND) and the distribution of CND subtypes

The prevalence of hospitalized children with CNDs was 2.22%, with PEM in 0.21%, obesity in 0.27%, and micronutrient deficiencies in 1.81%. The CND rate was highest among children aged 1 year to under 5 years and those living in the Northeast and South regions (Table 1). Moreover, the rate of hospitalized children with PEM increased from 1.53/1,000 cases in 2015 to 2.18/1,000 cases in 2019; the obesity rate also increased from 2.08/1,000 cases in 2015 to 2.61/1,000 cases in 2019; meanwhile, the proportion of patients with micronutrient deficiencies remained high throughout the study period (Supplementary Material 2).

**Figure 1.**
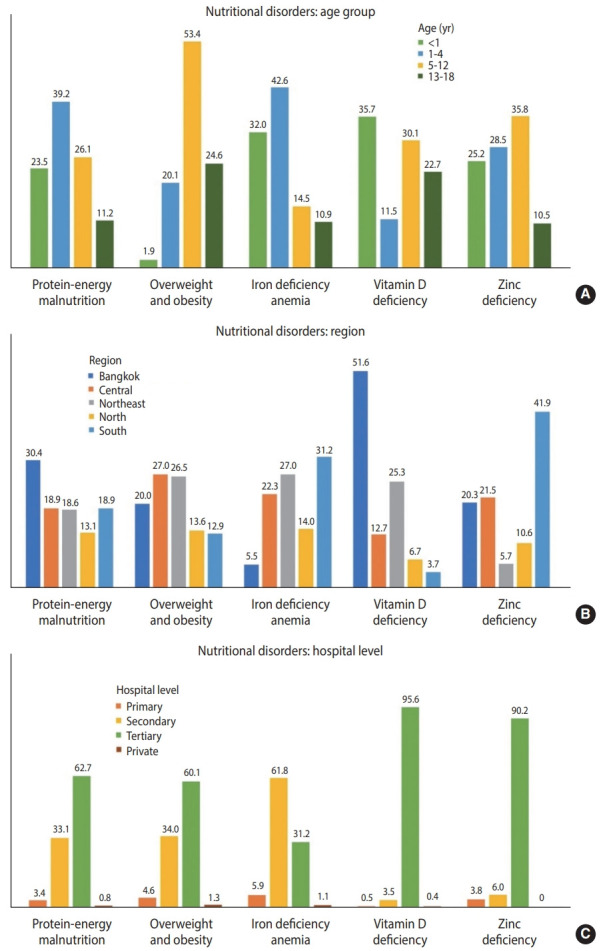
Distribution of nutritional disorders among hospitalized children aged 1 month to 18 years, stratified by (A) age, (B) hospital region, and (C) hospital level.

CND subtypes were analyzed based on patients’ age, region, and hospital level (Figure 1). PEM was more prevalent in patients aged 1 year to under 5 years (39.2%), those admitted in the Bangkok region (30.4%), and those who received tertiary care (62.7%). Obesity was particularly common among patients aged 5 years to under 13 years (53.4%), those living in the Central (27.0%) and Northeast regions (26.5%), and those who received tertiary care (60.1%).

Furthermore, 94,123 patients were diagnosed with micronutrient deficiencies. Iron deficiency anemia, vitamin D deficiency, and zinc deficiency were the three micronutrient deficiency subtypes that contributed to most cases. Iron deficiency anemia was detected in approximately 90,000 patients, the majority of whom were under the age of 5 years and lived in the Southern (31.1%) and Northeast (27.0%) regions. Vitamin D deficiency was predominantly evident in infants (35.7%) and those who were admitted to hospitals located in Bangkok. Meanwhile, zinc deficiency was mainly found in patients aged 5 years to under 13 years (35.8%) and those living in the South region (41.9%) (Figure 1 and Table 2).

### Comorbidities of children hospitalized with protein-energy malnutrition

Approximately 14,000 hospitalizations were recorded among children with a coded diagnosis of PEM, which had a rate of 1.59/1,000 hospitalizations in 2015-2019. The rate of PEM was highest in Bangkok (Supplementary Material 3). The most common comorbidities among patients with PEM as the principal diagnosis were disorders of fluid, electrolytes, and acid-base balance (E87, 12.1%), postprocedural disorders of the digestive system (K91, 10.6%), and disorders of mineral metabolism (E83, 7.1%) (Table 3). Conversely, the most common principal diagnoses among patients with PEM as a comorbidity were pneumonia (J18, 10.1%), other gastroenteritis and colitis of infectious and unspecified origin (A09, 7.7%), and bacterial pneumonia (J15, 5.3%) (Table 4).

### Clinical outcomes associated with hospitalized children with protein-energy malnutrition in tertiary-care hospitals

**Figure 2.**
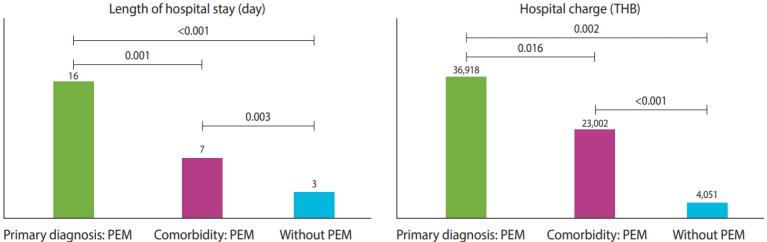
Estimated burden of hospitalized children with PEM at tertiary-care hospitals. PEM, protein-energy malnutrition; THB, Thai baht.

During the 5 fiscal years, 2,612,787 hospitalizations among 1,507,273 patients were documented in tertiary-care hospitals. The rate of PEM in tertiary-care hospitals was 3.81/1,000 hospitalizations, which was higher than those observed in primary (0.2/1,000) and secondary (1.9/1,000) hospitals. We analyzed patient outcomes in the following 3 groups: (1) patients with PEM as a principal diagnosis, (2) patients with PEM as a comorbidity, and (3) patients without a coded diagnosis of PEM. Children hospitalized with PEM, either as a principal diagnosis or a comorbidity, had significantly longer median LOS and higher hospital costs than children without PEM (Figure 2).

**Figure 3.**
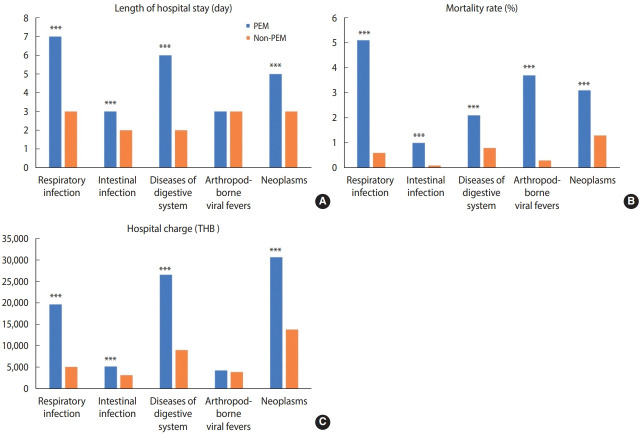
Difference between (A) length of hospital stay, (B) in-hospital mortality rates, and (C) hospital charges among inpatient discharges with and without a coded diagnosis of PEM by selected common diseases. PEM, protein-energy malnutrition; THB, Thai baht. ^***^p<0.001.

The common groups of primary diagnoses were respiratory infections, intestinal infections, digestive system disorders, and arthropod-borne viral fever. Patients with common diseases accompanied by PEM, except arthropod-borne viral fever, had significantly longer LOS, higher hospital costs, and higher in-hospital mortality rates than those without PEM. The most common primary diagnosis was respiratory infections, which predominantly affected children under the age of 5 years (77%). Moreover, the highest mortality rate in malnourished children who were admitted with respiratory tract infections was observed in children under the age of 5 years (74%). Regarding the association between PEM and neoplasms, patients with cancer with a coded diagnosis of PEM tended to have longer LOS, higher hospital costs, and higher in-hospital mortality rates than those without PEM (Figure 3).

## DISCUSSION

This study is the first to analyze a large dataset on malnutrition in hospitalized children in Thailand. In this study, the prevalence of nutritional disorders was 2.2%, and that of PEM was 0.2%. These rates were considerably lower than those reported in previous studies. Our prospective multicenter study revealed that 40% of hospitalized children had an abnormal nutritional status, including PEM or obesity, on admission; of these, 24% developed hospital-acquired malnutrition [4]. This discrepancy could be due to differences in the hospital settings, patients’ characteristics, the definition or method used to define malnutrition, and/or lack of identification. In the United States, 1.3% and 2.6% of hospitalized children had malnutrition in 2016 and 2018, respectively [7,8]. Malnutrition was found to be prevalent in 30% of pediatric inpatients in a single-center study in the United States; however, only 33% of malnourished patients were given a malnutrition-related ICD-10 code at discharge. A reimbursement calculation showed that coding for malnutrition resulted in an additional cost of US$27,664.70 to the hospital in 2 years [9]. Therefore, the true prevalence of nutritional disorders from ICD-10-CM codes was possibly underrepresented.

Although nutritional disorder coding did not reflect its true prevalence, the rates of PEM and obesity were found to have increased over the study period. This trend may have resulted from an increase in the awareness of healthcare providers; better practices, documentation, and coding systems; or a true increase in malnutrition prevalence. Additionally, advances in medical and surgical technology have led to a longer life expectancy for patients with congenital or chronic diseases [10-13]. These patients are at a high risk of developing malnutrition [14,15].

The major nutritional problems in hospitalized children aged under 5 years were PEM and iron deficiency anemia, whereas those in teenagers were obesity and vitamin D deficiency. These findings are consistent with global reports in a community setting that showed numerous children under the age of 5 years suffering from malnutrition and anemia. Moreover, overweight and obesity are also increasing rapidly worldwide [16]. Since this is the first study on hospitalized children using a national database, there were no data to compare the overall nutritional problems in the hospital settings. However, the South-East Asian Nutrition Survey (SEANUTS), which was conducted among children aged 0.5-12.9 years in 4 countries (Thailand, Indonesia, Malaysia, and Vietnam) in 2013, reported that the prevalence of childhood obesity was 1.1-2.2% in children aged 0.5-2.9 years, 5.0-8.2% in children aged 3.0-5.9 years, and 7.7-16.3% in children aged 6.0-12.9 years. Similarly, when compared to this study, overweight and obesity were mainly detected in young adults from SEANUTS. The rapid socioeconomic development, from agricultural to industrial, has resulted in changes in lifestyle, dietary patterns, food availability, social environment, and physical activity. These factors can contribute to the dramatic rise in obesity and noncommunicable diseases in younger children, particularly in urban areas [17-19].

Furthermore, using hemoglobin < 11.0 g/dL as a proxy indicator, the SEANUTS study found a prevalence of iron deficiency anemia between 9.0% and 18.4%, whereas using soluble transferrin receptor as an indicator revealed a prevalence between 32.3% and 38.9%. The highest prevalence of anemia was observed in infants and young children (0.5–5.9 years) [20], which was similar to the present study. Factors associated with iron deficiency anemia in young children include low socioeconomic status, prolonged breastfeeding, delayed age of starting iron-rich complementary foods, and low dietary iron intake [21]. Although studies demonstrating the cause of iron deficiency anemia in hospitalized children are scarce, some factors, such as parasitic infection, not eating meat or animal products, poor nutritional status, and acute lower respiratory tract infection, have been mentioned [22,23].

Furthermore, micronutrient deficiency, also known as “hidden hunger,” could occur in patients with normal weight and height status, those with acute illness, and those with chronic diseases that affect nutrient metabolism. Under-recognition and unavailability of specific laboratory testing for a definite diagnosis are the 2 possible reasons for micronutrient deficiencies to be under-reported.

In this study, PEM was more prevalent in Bangkok, the Southern region, and tertiary-care hospitals. The variation in PEM prevalence probably resulted from the differences in the causes and effects of multiple factors, including inappropriate dietary choices, food availability, socioeconomic status, limited healthcare access, and underlying chronic medical illnesses. Patients with PEM admitted with common infections had significantly longer hospital stays, higher hospital costs, and higher mortality rates than those without PEM. Previous studies have found that malnourished children are at increased risk of infection, longer hospital stays [3,24], and bear higher hospitalization costs [8]. A bidirectional relationship between malnutrition and infection, particularly diarrhea and pneumonia, has been established in developing countries [25]. Malnutrition was associated with a higher risk of mortality from diarrhea and acute lower respiratory infections in children under the age of 5 years [26]. Additionally, Kirolos et al. [27] reported that children with pneumonia who were moderately or severely malnourished were 2.0 times (95% confidence interval [CI], 1.6 to 2.6) and 4.6 times (95% CI, 3.7 to 5.9) more likely to die than children of normal weight. In a comparison between pre-2000 and post-2000 studies, the risk of death has remained high [27].

In terms of economics, Amaral et al. [28] found that the economic impact of disease-related malnutrition for patients with nutritional risk by multivariate estimation determinants of treatment cost was 20% higher than the average. Moreover, in previous Thai studies, malnourished children had significantly longer LOS and higher total hospital costs than non-malnourished patients [4,29].

Our study demonstrated that inpatient care in terms of nutrition may be improved through nutritional disorder awareness and a coding system for reimbursement. Nutritional issues are often not perceived as a priority. Physicians usually focus on management and treatment for acute conditions, ignoring the risk of malnutrition or nutritional-related problems. Although weight and height assessments are simple, they may be challenging for severely ill patients.

According to the present study, the prevalence of malnutrition differed between tertiary-care hospitals and government hospitals in all regions. Tertiary-care hospitals reported a higher rate of PEM than all NHSO data in every region. The children hospitalized in tertiary-care hospitals tended to have more chronic and complicated illnesses related to inflammation, alterations in energy and metabolism, decrease in food intake, and increase in nutrient loss; these conditions contribute to malnutrition risk and compromise recovery [30,31]. These differences among patients in baseline characteristics and the etiology of patients could lead to diverse undernutrition rates. Furthermore, medical teaching programs at tertiary-care centers may contribute to the completion of all diagnoses of patients; thus, the rate of undernutrition may increase, thereby bringing it closer to the “true” prevalence.

The DRG is the main determinant of inpatient-care expenditures for reimbursement. To code for a high payment rate, the coders tend to code the principal diagnosis and procedures, allowing for higher reimbursement, rather than the actual diagnosis. However, some diagnoses and complications are missed because of low or no financial benefit.

Unfortunately, in Thailand, other nutritional interventions have no corresponding codes for the repayment of hospital costs. Only parenteral nutrition can be reimbursed using the code 99.15 [32]. In addition, hospital policy may not facilitate and support nutrition prevention and management workflow. Considering that the hospital costs of nutritional interventions are non-refundable tangible losses, merely a small budget is allocated for investing in nutritional care. Furthermore, some physicians are unsure about providing nutritional support and are hesitant to implement daily nutritional care. To combat pediatric hospital malnutrition, pediatricians, general physicians, and healthcare providers need to receive nutritional education and empowerment. Moreover, nutritional screening and assessment should be incorporated in the training program for medical students and pediatric residents, as well as a refresher program for graduated personnel. In this way, healthcare providers may be able to identify nutritional problems in hospitalized children.

In Thailand, most university and tertiary-care hospitals have clinical nutrition teams, including pediatric nutrition specialists, dietitians, nutrition nurse specialists, and pharmacists. The nutrition care team is variable across different hospital settings, but is likely to consist of at least a clinician, nurse, and dietitian. However, in many hospitals, under-recognition or inadequate screening lead to the delay of nutritional consultation, assessment, and intervention, as well as a lack of continued monitoring of the nutritional response. Additionally, malnutrition continues to be identified and managed predominantly by the clinical nutrition team, while the primary care team may not be involved in this process. The effective management of nutritional disorders in hospitals requires collaboration among all disciplines involved in patient care. Nutrition specialists should be included in a patient’s care team. Moreover, nutritional screening using valid screening tools should be implemented in routine care, and nutritional issues should be included in the daily problem list.

Our study investigated the national prevalence of nutritional disorders, which were stratified by age group, region, and hospital levels, and determined the burden of hospitalized children with PEM. PEM and obesity in Thailand showed increasing trends during the study period. However, this study has some potential limitations. First, although this study presented the national data, the validity of the findings could be a limitation. Although the overall prevalence of PEM was lower than expected, the prevalence of PEM was much higher in tertiary-care settings. This could be explained by the higher severity of diseases and more careful evaluation of nutritional status in the tertiary-care setting. The factors determining data reliability include the absence of routine nutritional assessments, failure to recognize nutritional problems, and incorrect coding. However, the data depict the importance of nutritional disorders and raise awareness of thorough evaluation of nutritional disorders in hospitalized pediatric patients.

Some micronutrient deficiencies, such as vitamin A, vitamin E, and zinc deficiencies, need special laboratory testing, which is available only at some university hospitals and is not widely used in clinical practice. Second, the data did not contain information on the precise timing of diagnoses; thus, we could not clarify whether nutritional disorders developed before or during hospitalization. Third, the ICD-10-CM is available only for principal diagnoses and comorbidities. As mentioned, the codes of nutritional interventions and some nutritional-related problems, such as refeeding syndrome, are still unavailable.

These informative findings from our study have led to the formulation of the following proposed actions: (1) nutritional education for healthcare professionals should be implemented to increase awareness and achieve the benefits of nutritional support in hospitalized pediatric patients; (2) nutritional screening tools should be developed and implemented to identify patients at risk for malnutrition, followed by assessment and proper treatment to improve the hospital outcomes; (3) primary physicians should add nutritional issues to their interdisciplinary approach to patient care and include nutrition specialists in teams; (4) collaborative nutritional support should be established, especially in high-risk groups, as a basic health service; (5) specific International Classification of Diseases codes should be made available to indicate nutritional interventions including oral nutritional support, enteral nutrition, and parenteral nutrition; and (6) nutritional services and interventions should be reimbursed to achieve proper health care and better outcomes.

In conclusion, malnutrition is common among hospitalized children; it increases morbidity, mortality, LOS, and hospital costs and exerts long-term effects on growth and development. Access to nutritional screening tools and education in medical schools are recommended for early detection and interventions. Specific International Classification of Diseases codes for nutritional care services and interventions, such as nutritional counseling, special diets, oral nutritional supplements, and enteral and parenteral nutrition, should also be established. Furthermore, nutritional interventions should be reimbursed, along with the provision of nutritional education and empowerment of healthcare providers, to improve hospital care services and patient outcomes.

## Figures and Tables

**Table 1.** Diagnosis of hospitalized children with a coded diagnosis of nutritional disorders based on ICD-10-CM codes in a nationwide inpatient sample, 2015-2019

Characteristics		Inpatient	Patients with nutritional disorders	Protein-energy malnutrition	Overweight and obesity	Micronutrient deficiencies
Total cases		5,188,033	115,254 (2.2)	11,128 (0.2)	14,107 (0.3)	94,123 (1.8)
Fiscal year						
	2015	1,359,734	21,601 (1.6)	2,076 (0.1)	2,829 (0.2)	18,868 (1.4)
	2016	1,420,800	24,434 (1.7)	2,266 (0.2)	2,869 (0.2)	21,756 (1.5)
	2017	1,345,542	23,111 (17.2)	2,015 (0.1)	3,054 (0.2)	20,366 (1.5)
	2018	1,378,152	23,061 (1.7)	1,815 (0.1)	3,271 (0.2)	20,294 (1.5)
	2019	1,358,121	23,047 (1.7)	2,956 (0.2)	3,541 (0.3)	18,868 (1.4)
Age (yr)						
	<1	241,397	31,938 (13.2)	2,616 (1.1)	257 (0.1)	30,202 (12.5)
	1-5	1,379,244	44,854 (3.2)	4,362 (0.3)	2,838 (0.2)	39,251 (2.8)
	5-13	2,066,184	23,798 (1.1)	2,904 (0.1)	7,536 (0.4)	14,205 (0.7)
	13-18	1,501,208	14,664 (1.0)	1,246 (0.1)	3,476 (0.2)	10,465 (0.7)
Region						
	Bangkok	385,946	12,117 (3.1)	3,383 (0.9)	2,814 (0.7)	6,352 (1.6)
	Central	1,317,916	25,822 (2.0)	2,113 (0.2)	3,816 (0.3)	20,823 (1.6)
	Northeast	1,742,680	30,840 (1.8)	2,070 (0.1)	3,744 (0.2)	25,286 (1.4)
	North	837,826	15,742 (1.9)	1,458 (0.2)	1,918 (0.2)	12,927 (1.5)
	South	903,665	30,733 (3.4)	2,104 (0.2)	1,815 (0.2)	28,735 (3.2)
Hospital level						
	Primary care	NA	6,379	377	636	5,460
	Secondary care	NA	62,853	3,683	4,798	56,111
	Tertiary care	1,507,273	44,765 (3.0)	6,980 (0.5)	8,486 (0.6)	31,524 (2.1)
	Private care	NA	1,257	88	187	1,028

Values are presented as number or number (%). ICD-10-CM, International Classification of Diseases, 10th revision, Clinical Modification; NA, not available.

**Table 2.** Diagnosis of hospitalized children with a coded diagnosis of micronutrient deficiencies based on ICD-10-CM codes in a nationwide inpatient sample, 2015-2019

Characteristics		Inpatient, n	Micronutrient deficiencies	Iron deficiency anemia: D50	Vitamin D deficiency: E55	Zinc deficiency: E60
Total cases in 5 fiscal year		5,188,033	94,123 (1.81)	89,925 (1.73)	2,060 (0.04)	650 (0.01)
Fiscal year						
	2015	1,359,734	18,868 (1.39)	17,738 (1.30)	393 (0.03)	226 (0.02)
	2016	1,420,800	21,756 (1.53)	20,745 (1.46)	403 (0.03)	172 (0.01)
	2017	1,345,542	20,366 (1.51)	19,458 (1.45)	521 (0.04)	82 (0.01)
	2018	1,378,152	20,294 (1.47)	19,308 (0.14)	545 (0.04)	170 (0.01)
	2019	1,358,121	18,868 (1.39)	17,938 (1.32)	624 (0.05)	119 (0.01)
Age (yr)						
	<1	241,397	30,202 (12.51)	28,771 (11.92)	735 (0.30)	164 (0.07)
	1-5	1,379,244	39,251 (2.85)	38,356 (2.78)	237 (0.02)	185 (0.01)
	5-13	2,066,184	14,205 (0.69)	13,025 (0.63)	621 (0.03)	233 (0.01)
	13-18	1,501,208	10,465 (0.70)	9,773 (0.65)	467 (0.03)	68 (0.00)
Region						
	Bangkok	385,946	6,352 (1.65)	4,910 (1.27)	1,063 (0.28)	132 (0.03)
	Central	1,317,916	20,823 (1.58)	20,016 (1.52)	261 (0.02)	140 (0.01)
	Northeast	1,742,680	25,286 (1.45)	24,257 (1.39)	522 (0.03)	37 (0.00)
	North	837,826	12,927 (1.54)	12,572 (1.50)	137 (0.02)	69 (0.01)
	South	903,665	28,735 (3.18)	28,170 (3.12)	77 (0.01)	272 (0.03)

Values are presented as number (%). ICD-10-CM, International Classification of Diseases, 10th revision, Clinical Modification.

**Table 3.** Comorbidities of hospitalized children with protein-energy malnutrition as a coded principal diagnosis

ICD-10 codes and titles	%
K91.2 Postsurgical malabsorption, not elsewhere classified	10.4
E87.6 Hypokalemia	4.2
E87.2 Acidosis	3.4
E87.1 Hypo-osmolality and hyponatremia	3.2
E83.4 Disorders of magnesium metabolism	2.9
D50.9 Iron deficiency anemia, unspecified	2.3
E86 Volume depletion	2.3
D64.9 Anemia, unspecified	2.0
E88.3 Tumor lysis syndrome	1.8
A09.9 Gastroenteritis and colitis of unspecified origin	1.7

ICD-10, International Classification of Diseases, 10th revision.

**Table 4.** Principal diagnoses of hospitalized children with protein-energy malnutrition as a coded comorbidity

ICD-10 codes and titles	%
J18.9 Pneumonia, unspecified	7.6
A09.9 Gastroenteritis and colitis of unspecified origin	6.0
J15.9 Bacterial pneumonia, unspecified	3.3
J20.9 Acute bronchitis, unspecified	2.5
J69.0 Pneumonitis due to food and vomit	1.9
A09.0 Other and unspecified gastroenteritis and colitis of infectious origin	1.7
C91.0 Acute lymphoblastic leukemia	1.7
J12.9 Viral pneumonia, unspecified	1.5
J180 Bronchopneumonia, unspecified	1.4
A08.4 Viral intestinal infection, unspecified	1.2

ICD-10, International Classification of Diseases, 10th revision.

## SUPPLEMENTARY MATERIALS

Supplementary Material 1. ICD-10-CM diagnosis codes for nutritional disorders

Supplementary Material 2. Prevalence of malnutrition with coded diagnoses of nutritional disorders in hospitalized children by year (per 1000 cases)

Supplementary Material 3. Rate of hospitalized children with a coded diagnosis of protein-energy malnutrition per 1000 admissions (total NHSO vs. tertiary care)
