# Supplementary material for: Epidemiological data on nutritional disorders and outcomes in hospitalized Thai children: an analysis of data from the National Health Database 2015-2019
Source: Epidemiol Health. 2022 May 16;44:e2022047. doi: 10.4178/epih.e2022047 (PMC9684013; doi:10.4178/epih.e2022047)
Supplement: Supplementary Material 1. — ICD-10-CM diagnosis codes for nutritional disorders [file epih-44-e2022047-suppl1.docx]

**Supplementary Material 1. ICD-10-CM diagnosis codes for nutritional disorders**

| **Categories of nutritional disorders** | **ICD-10 Code** | **ICD-10 Title** |
| --- | --- | --- |
| Protein-energy malnutrition | E40 | Kwashiorkor |
|  | E41 | Nutritional marasmus |
|  | E42 | Marasmic kwashiorkor |
|  | E43 | Unspecified severe protein-energy malnutrition |
|  | E44 | Protein-energy malnutrition of moderate and mild degree |
|  | E45 | Retarded development following protein-energy malnutrition |
|  | E46 | Unspecified protein-energy malnutrition |
| Overweight and obesity | E66 | Obesity |
| Micronutrient deficiencies | D50 | Iron deficiency anemia |
|  | D51 | Vitamin B12 deficiency anemia |
|  | D52 | Folate deficiency anemia |
|  | D53 | Other nutritional anemia |
|  | E50 | Vitamin A deficiency |
|  | E51 | Thiamine deficiency |
|  | E52 | Niacin deficiency |
|  | E53 | Riboflavin deficiency |
|  | E54 | Ascorbic acid deficiency |
|  | E55 | Vitamin D deficiency |
|  | E56 | Deficiency of vitamin K |
|  | E58 | Dietary calcium deficiency |
|  | E60 | Dietary zinc deficiency |
|  | E61 | Deficiency of other nutrient elements |
|  | E63 | Other nutritional deficiency |

ICD-10-CM, International Classification of Diseases, Tenth Revision. Clinical Modification
