# Supplementary material for: Epidemiological data on nutritional disorders and outcomes in hospitalized Thai children: an analysis of data from the National Health Database 2015-2019
Source: Epidemiol Health. 2022 May 16;44:e2022047. doi: 10.4178/epih.e2022047 (PMC9684013; doi:10.4178/epih.e2022047)
Supplement: Supplementary Material 3. — Rate of hospitalized children with a coded diagnosis of protein-energy malnutrition per 1000 admissions (total NHSO vs. tertiary care) [file epih-44-e2022047-suppl3.docx]

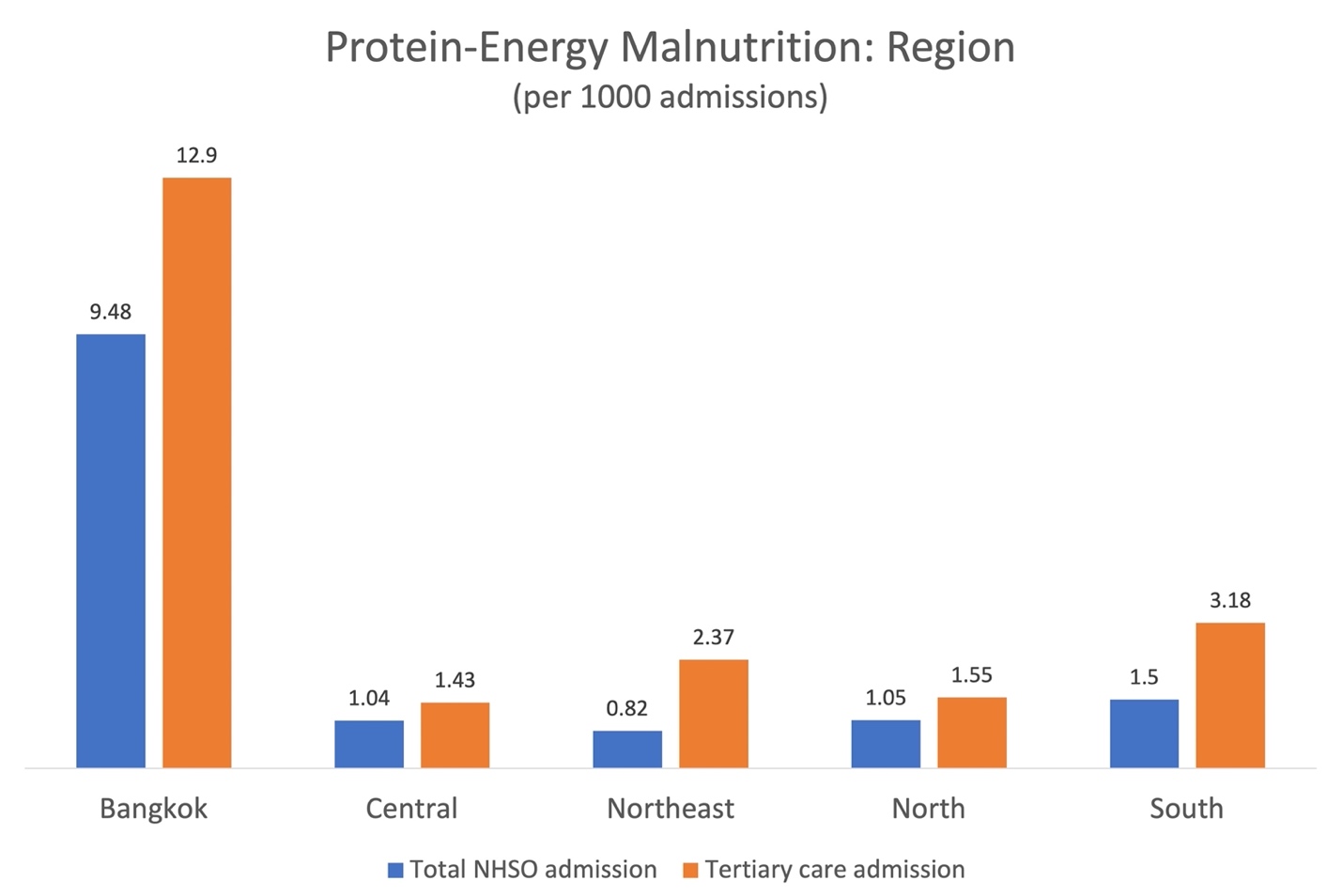


**Supplement Material 3. Rate of hospitalized children with a coded diagnosis of protein-energy malnutrition per 1000 admissions (total NHSO vs. tertiary care)**
